# Ametryn and Clomazone Disrupt Mitochondrial Bioenergetics in Rat Liver: Evidence for Inhibition of Complexes I and II and ATP Synthase

**DOI:** 10.3390/toxics13090784

**Published:** 2025-09-16

**Authors:** Heberth Paulo dos Santos Silva, Camila Ortiz, Camila Araújo Miranda, Paulo Francisco Veiga Bizerra, Carlos Manuel Palmeira, Fábio Erminio Mingatto

**Affiliations:** 1 Department of Animal Science, College of Agricultural and Technological Sciences, São Paulo State University (Unesp), Dracena 17915-899, SP, Brazil; heberth.paulo@unesp.br (H.P.d.S.S.); camila.ortiz@etec.sp.gov.br (C.O.); camila.miranda@unesp.br (C.A.M.); p.f.veigabizerra@hotmail.com (P.F.V.B.); 2 Department of Life Sciences and Center for Neurosciences and Cell Biology, University of Coimbra, 3004-531 Coimbra, Portugal; palmeira@uc.pt

**Keywords:** pesticides, toxicology, liver, mitochondrial dysfunction, adenosine triphosphate

## Abstract

Ametryn (AMT) and clomazone (CLZ) are commonly used herbicides frequently detected in food and water, raising concerns about potential health risks. This study investigated whether AMT and CLZ impair mitochondrial bioenergetics, a key mechanism linked to hepatotoxicity. Mitochondria were isolated from rat liver and incubated with AMT or CLZ (50–200 µM) to assess respiration, membrane potential (Δψ), ATP production, and the activities of respiratory chain complexes and ATP synthase. Both herbicides significantly inhibited state 3 (ADP-stimulated) respiration with glutamate plus malate, without altering state 4 (basal) respiration. Concentrations above 100 µM reduced Δψ and ATP synthesis in glutamate plus malate or succinate-energized mitochondria. Enzymatic assays revealed inhibition of complex I by both herbicides, complex II by CLZ, and ATP synthase by both. These results highlight mitochondrial oxidative phosphorylation disruption by AMT and CLZ; however, further in situ and in vivo studies are necessary to fully understand their hepatotoxic potential.

## 1. Introduction

Ametryn (AMT) is an *s*-triazine herbicide widely used in agriculture to control weeds in cultures of corn, sugarcane, and other crops. The compound works by inhibiting the electron transport chain, binding to the D1 proteins of photosystem II, which prevents photosynthesis and plant growth [1]. AMT has been detected in agricultural soils at concentrations up to 2.58 µg/g dry weight and in irrigation waters reaching 0.1 µg/L [2]. AMT contamination also have been reported in surface waters in Brazil (0.0001–0.148 μg/L [3]; 0.006–1.1 μg/L [4]; 10–60 μg/L [5]), Costa Rica (0.1–20 μg/L [6]) and United States (<0.003–12 µg/L [7]), and groundwater sites in Hawaii (0.7 μg/L [8]) and United States (0.021–54 µg/L [7]). AMT can be present in certain foods as pineapple (0.16 mg/kg dry weight [9] and ≤0.02 mg/kg dry weight [10]) and carrot (≤0.02 mg/kg dry weight [10]). A detection method for AMT residues in rice, apple, banana, and eggplant achieved a limit of detection (LOD) as low as 0.01 mg/kg dry weight, although actual residue levels in samples were not specified [11]. The acceptable daily intake for AMT is 0.072 mg/kg body weight, based on long-term toxicological studies [8]. To protect public health, regulatory agencies have also established maximum residue levels in food commodities; for instance, the United States Environmental Protection Agency has set tolerances of 0.25 ppm for bananas, 0.10 ppm for corn forage, and 0.05 ppm for corn grain, pineapple and sugarcane [12]. In drinking water, the Australian National Health and Medical Research Council recommends a guideline value of 0.07 mg/L, ensuring that total exposure remains below the ADI for an average adult [13].

Clomazone (CLZ) is a herbicide belonging to the class of isoxazolidinones and is widely used against weeds in a variety of crops, such as soybean, peanut, rice, and sugarcane. CLZ works by inhibiting weed growth through disruption of carotenoid biosynthesis, which is essential for plant growth and development [14]. CLZ residues were detected in soils at varying concentrations and depths, ranging from 1 to 218 μg/kg dry weight, with an average concentration of 56 μg/kg dry weight [15] and in drinking, surface and spring waters from Brazil, China, Costa Rica, and England, with concentrations ranging from 0.01 to 22 μg/L [3,16,17,18,19,20,21,22]. Residue analysis has shown CLZ levels < 0.02 mg/kg dry weight in food commodities, such as dry beans [23], potatoes and oilseed rape [24]. From a regulatory perspective, the acceptable daily intake for clomazone is 0.13 mg/kg body weight, and the acute reference dose is 2.5 mg/kg [25].

The liver is one of the organs most affected by exposure to AMT. Studies have shown that AMT can induce significant hepatic damage in different biological models. For instance, in isolated hepatocytes and primary cultured hepatocytes, exposure to the herbicide was found to increase the release of lactate dehydrogenase (LDH), a marker of cytotoxicity [26]. Important changes in liver structure were observed following subchronic and chronic oral exposure of rabbits, mice, dogs, and rats to AMT [27]. Studies also have shown that CLZ can induce hepatotoxicity in animals, including structural alterations in hepatocytes, such as swelling and lysis of mitochondrial cristae, as well as oxidative damage [28,29,30,31].

Because it lies between the gastrointestinal tract and the systemic circulation, the liver serves as a major hub for metabolism and xenobiotic detoxification, making it one of the primary target organs for chemical exposure [32]. Liver damage cannot be regarded as a single entity; rather, the observed lesions depend not only on the type of substance involved, but also on its concentration and the duration of exposure. The impact of industrial by-products or residues on human and animal liver function, particularly through environmental contamination, has been well-documented [33]. These pollutants can cause a range of health issues, such as necrosis, steatosis, cirrhosis, and carcinoma, by disrupting normal cellular processes [34,35,36,37].

Mitochondria generate the majority of cellular ATP necessary for homeostasis, and evidence indicates that they represent a primary and vulnerable target for the effects of drugs, toxins, and reactive metabolites produced by cytochrome P450 enzymes, often leading to mitochondrial dysfunction and cytotoxicity [38,39]. Adverse effects on mitochondria can occur via both direct and indirect mechanisms, including alterations in the electron transport chain and oxidative phosphorylation, ultimately leading to ATP depletion and cell death [40,41,42,43]. Additionally, the mitochondrial electron transport chain (ETC) is a major intracellular source of reactive oxygen species (ROS), particularly at complexes I and III [44]. In this way, Chen et al. (2013) [45] demonstrated that microcystin-LR impairs the electron transport chain, triggering increased ROS generation, mitochondrial DNA damage, and cytotoxicity, reinforcing the close link between disruption of oxidative phosphorylation and ROS-mediated injury. Previous studies have shown that both AMT and CLZ can promote oxidative stress, with mitochondria acting as a key source and target of redox imbalance. In vivo exposure to AMT in Wistar rats increased oxidative stress markers and modulated antioxidant defenses [e.g., decreased superoxide dismutase (SOD) activity with compensatory changes in Mn-SOD transcripts] consistent with mitochondria-derived ROS signaling [46], while AMT also induced oxidative stress and developmental toxicity in zebrafish embryos [47]. For CLZ, oxidative injury has been reported in human erythrocytes in vitro (increased oxidative stress and AChE inhibition [48]) and in fish (*Rhamdia quelen*), CLZ exposure altered several oxidative stress indicators, including increased thiobarbituric acid reactive substances and protein carbonylation in liver, with stimulation of antioxidant defenses (SOD, glutathione *S*-transferase, ascorbic acid and non-protein thiols levels increase [49]).

Mitochondrial energetic function is commonly evaluated through measurements of oxygen consumption, ATP synthesis, and mitochondrial membrane potential; however, the activities of specific enzymes can also provide valuable insights into the integrity of the ETC [50]. In the category of oxidases, the activities of NADH oxidase, succinate oxidase, and cytochrome c oxidase (complex IV) can be measured. NADH oxidase activity reflects the capacity to oxidize NADH and feed electrons into the ETC [51]. Succinate oxidase activity, typically assessed using succinate as a substrate in the presence of rotenone, reports on electron transfer via complex II [52]. Cytochrome c oxidase catalyzes the terminal electron transfer to molecular oxygen, directly indicating the efficiency of respiratory function and ATP production [53]. For dehydrogenases, NADH dehydrogenase reflects electron entry from NADH into the ETC, whereas succinate dehydrogenase links the tricarboxylic acid cycle with the ETC through FADH_2_-mediated electron transfer [51,54]. Together, these measurements allow a comprehensive assessment of mitochondrial bioenergetic capacity by probing both upstream electron entry and downstream oxidative steps.

Mitochondrial dysfunction has been associated with AMT and CLZ cytotoxicity [30,55] but the precise mechanism involved remains unknown. The main hypothesis is that AMT and CLZ interfere with mitochondrial bioenergetics, specifically targeting respiratory chain complexes, leading to reduced ATP production and membrane potential. Despite belonging to different chemical classes and possessing distinct modes of action, AMT and CLZ are often employed in combination in commercial formulations, which enhance their efficacy in managing invasive plants [56,57,58]. Additionally, environmental studies have detected the simultaneous presence of AMT and CLZ in soils and water bodies near agricultural areas [59,60,61,62], raising concerns about their toxic effects on non-target organisms. Considering these factors, the present study investigates the effects of AMT and CLZ on bioenergetic functions in mitochondria isolated from rat liver, evaluating their impact on respiration, membrane potential, ATP production, and mitochondrial enzyme activities.

## 2. Materials and Methods

### 2.1. Chemicals

AMT (Catalog Number: 45321; purity = 100%) and CLZ (Catalog Number: 46120; purity = 100%) were purchased from Sigma-Aldrich (St. Louis, MO, USA). All other reagents were commercial products of standard chemical grade. Ultrapure water (Direct Q3, Millipore, Bedford, MA, USA) was used. All other reagents were commercially available of standard chemical grade. Ultrapure water (Direct Q3, Millipore, Bedford, MA, USA) was used to prepare the solutions. AMT and CLZ were dissolved in dimethyl sulfoxide (DMSO), and the final concentration of DMSO in the assay medium did not exceed 0.1% (*v*/*v*) in any experiment, a concentration that had no detectable effect on mitochondrial function.

### 2.2. Animals

Male Wistar rats with an average body weight of approximately 200 g were used in this study. The rats were housed under standard laboratory conditions (temperature, 23 ± 3 °C; humidity, 55 ± 15%; light/dark cycle, 12 h:12 h), with a maximum of four per cage, and had ad libitum access to water and food. Animals were supplied by the Central Animal Facility of São Paulo State University (Unesp), Campus of Botucatu, SP, Brazil. All experimental procedures were approved by the Ethical Committee for the Use of Laboratory Animals of the São Paulo State University (Unesp), Campus of Dracena, SP, Brazil (Approval numbers 13/2018.SP and 16/2022.R1).

### 2.3. Experimental Design

In all experiments, the herbicides were used individually (i.e., not in combination). The exact incubation duration and temperature varied depending on the specific assay performed and are detailed in the corresponding Section 2. For each experiment, mitochondria were incubated under controlled conditions (time, temperature, buffer composition, and pH specified in each procedure). Technical replicates were performed in triplicate using four–six independent mitochondrial preparations to ensure reproducibility and robustness of the findings.

### 2.4. Isolation and Disruption of Rat Liver Mitochondria

Mitochondria were isolated by conventional differential centrifugation [63]. Rats were anesthetized by brief inhalation of diethyl ether and subsequently euthanized by decapitation. The liver was promptly excised, sliced, and rinsed three times with 50 mL of ice-cold medium containing 250 mM sucrose, 1 mM EGTA, and 10 mM HEPES-KOH (pH 7.2). The sliced tissue was then homogenized in a cold bath using a Potter-Elvehjem homogenizer, with three 15 s homogenization cycles performed at 1 min intervals. After centrifuging the homogenate for 5 min at 770× *g*, the supernatant was centrifuged again for 10 min at 9800× *g*. The pellet was centrifuged at 4500× *g* for 15 min after being suspended in 10 mL of media containing 250 mM sucrose, 0.3 mM EGTA, and 10 mM HEPES-KOH (pH 7.2). The final mitochondrial pellet was used within 3 h after being suspended in 1 mL of a medium containing 250 mM sucrose and 10 mM HEPES-KOH (pH 7.2). The biuret assay was used to measure the concentration of mitochondrial protein using bovine serum albumin (BSA) as the standard [64].

A heat shock treatment, with three successive cycles of freezing in liquid nitrogen and thawing in a water bath heated to 37 °C, was used to obtain disrupted mitochondria. Following this process, the protein concentration was measured to account for any potential protein loss resulting from freeze–thaw cycles. Membrane fragments were maintained at 4 °C and utilized for mitochondrial enzyme activity assays within 3 h.

### 2.5. Mitochondrial Respiration Assay

Mitochondrial respiration was assessed using a Clark-type oxygen electrode (Strathkelvin Instruments Limited, Glasgow, Scotland, UK), with respiratory parameters measured following the methods of Chance and Williams (1956) [65] using Strathkelvin Oxygen 782 System software (version 3.0, 2005). Mitochondrial protein (1 mg) was added to a 1 mL respiration buffer composed of 125 mM sucrose, 65 mM KCl, and 10 mM HEPES-KOH (pH 7.4), supplemented with 0.5 mM EGTA and 10 mM K_2_HPO_4_, at 30 °C. For the assessment of complex I–linked respiration, oxygen consumption was measured in the presence of 5 mM glutamate and 5 mM malate, which provide NADH via the tricarboxylic acid cycle and feed electrons into the respiratory chain through complex I (NADH:ubiquinone oxidoreductase). For evaluation of complex II-linked respiration, mitochondria were energized with 5 mM succinate (+50 nM rotenone) in the presence of 50 nM rotenone to block reverse electron flow to complex I. Succinate donates electrons via succinate dehydrogenase (complex II), bypassing complex I and feeding electrons directly into the ubiquinone pool. Herbicides were added to the reaction medium containing mitochondria and incubated for 2 min prior to the addition of respiratory substrates. The total recording time after substrate addition was 4 min.

State 3 respiration (ADP-stimulated) was induced by the addition of ADP (400 nmol) in the presence of the substrates, and the subsequent maximal ADP-coupled oxygen consumption rate was recorded. State 4 (resting) respiration was determined after complete phosphorylation of ADP to ATP (return to basal rate). Only mitochondrial preparations with Respiratory Control Ratio (RCR = state 3/state 4) higher than 4.0 were used in the experiments.

### 2.6. Estimation of Mitochondrial Membrane Potential (*Δψ*)

The mitochondrial membrane potential (Δψ) was assessed spectrofluorometrically with an RF-5301 PC fluorescence spectrophotometer (Shimadzu, Tokyo, Japan), using excitation and emission wavelengths of 495 and 586 nm, respectively. Safranin O (10 µM) was employed as the fluorescent probe [66]. Mitochondria (2 mg protein) were incubated at 30 °C in 2 mL of medium containing 125 mM sucrose, 65 mM KCl, 10 mM HEPES-KOH (pH 7.4), and 0.5 mM EGTA. Respiration was supported either by 5 mM glutamate plus 5 mM malate or by 5 mM succinate in the presence of 50 nM rotenone. Changes in relative fluorescence units (RFU) were normalized to the control condition (mitochondria without herbicide), which was considered to represent 100% of the formed membrane potential. At the end of each measurement (total recording time: 250 s), 1 µM carbonyl cyanide 3-chlorophenylhydrazone (CCCP) was added to fully dissipate the proton motive force, providing the reference for Δψ = 0. Membrane potential values for treatments were then expressed as a percentage of the control value.

### 2.7. ATP Quantification

ATP levels in mitochondria were quantified using the firefly luciferin-luciferase assay system [67]. Mitochondria (1 mg protein), energized with 5 mM glutamate plus 5 mM malate or 5 mM succinate in the presence of 50 nM rotenone, were incubated with the herbicides at 30 °C in 1 mL of medium containing 125 mM sucrose, 65 mM KCl, 10 mM HEPES-KOH (pH 7.4), 0.5 mM EGTA, and 10 mM K_2_HPO_4_. The reaction was initiated upon substrate addition and terminated after 10 min by centrifugation at 9000× *g* for 5 min at 4 °C, and the pellet was treated with 1 mL ice-cold 1 M HClO_4_. Subsequently, the supernatant was centrifuged at 14,000× *g* for 5 min at 4 °C, and 100 µL aliquots of the supernatant were neutralized with 5 M KOH and suspended in 100 mM TRIS-HCl (pH 7.8) to a final volume of 1 mL. After centrifugation at 15,000× *g* for 15 min, the supernatant was analyzed using the ATP Bioluminescent Assay Kit (FLAA, Sigma-Aldrich, St. Louis, MO, USA) following the manufacturer’s instructions and measured with a SIRIUS Luminometer (Berthold, Pforzheim, Germany). The ATP concentration was estimated by interpolation of the sample data into a standard curve ranging from 2 × 10^−10^ to 2 × 10^−7^ M ATP.

### 2.8. Determination of Enzymatic Activities

Mitochondrial oxidase activities were determined according to Bracht et al. (2003) [68], using a Clark-type oxygen electrode (Strathkelvin Instruments Ltd., Glasgow, Scotland, UK). Mitochondrial protein (0.5 mg) was added to 1 mL of a 20 mM Tris-HCl buffer (pH 7.4) at 30 °C. NADH oxidase activity was measured with 1 mM NADH, succinate oxidase was measured with 10 mM succinate, and cytochrome *c* oxidase was measured in the presence of 0.2 mM TMPD and 5 mM ascorbate. Herbicides were added to the reaction medium containing mitochondria and incubated for 2 min prior to the addition of respiratory substrates. The total recording time after substrate addition was 4 min.

The activity of NADH and succinate dehydrogenase was measured spectrophotometrically according to Singer (1974) [52], using a DU-800 spectrophotometer (Beckman Coulter, Fullerton, CA, USA). NADH dehydrogenase activity was measured in a reaction medium containing 50 mM NaH_2_PO_4_ (pH 7.4), 2 mM EDTA, 0.17 mM NADH, and 0.1 mg/mL mitochondrial protein in a final volume of 1.5 mL at 28 °C. The reaction was initiated by adding 0.6 mM potassium ferricyanide. Succinate dehydrogenase activity was assessed under similar conditions, using 50 mM NaH_2_PO_4_ (pH 7.4), 2 mM EDTA, 10 mM succinate, and 1 mg/mL mitochondrial protein, with 0.6 mM potassium ferricyanide to start the reaction; data were recorded for 1 min. In both assays, the reduction in ferricyanide was monitored as a decrease in absorbance at 420 nm, and enzyme activity was expressed as μmol·min^−1^·mg^−1^ protein, using a molar extinction coefficient of 1.04 mM^−1^·cm^−1^.

Mitochondrial ATPase activity was determined in both intact-uncoupled and freeze–thaw-disrupted mitochondria following the method of Bracht et al. (2003) [68], with modifications. Intact mitochondria (1 mg protein/mL) were incubated at 37 °C in 0.5 mL of medium containing 125 mM sucrose, 65 mM KCl, 10 mM HEPES-KOH (pH 7.4), 0.2 mM EGTA, and 5 mM ATP, in the presence of 1 μM CCCP. For disrupted mitochondria, the medium consisted of 20 mM Tris–HCl (pH 7.4). The reaction was initiated by adding 5 mM ATP and terminated after 20 min with ice-cold 5% trichloroacetic acid. ATPase activity was determined by measuring the released inorganic phosphate according to Fiske and Subbarow (1925) [69] at 700 nm using a DU-800 spectrophotometer (Beckman Coulter, Fullerton, CA). Results were expressed as nmol Pi·min^−1^·mg protein^−1^. Sensitivity to oligomycin (1 μg/mL) was assessed in all mitochondrial preparations.

### 2.9. Statistical Analysis

Data are presented as mean ± the standard error of the mean (SEM). Statistical analysis was conducted using GraphPad Prism v 9.0 (GraphPad Software, San Diego, CA, USA), applying one-way analysis of variance (ANOVA) followed by Dunnett’s post hoc test for multiple comparisons. Differences were considered statistically significant at *p* < 0.05.

## 3. Results

### 3.1. Effects of AMT and CLZ on Mitochondrial Respiration

**Figure 1:**
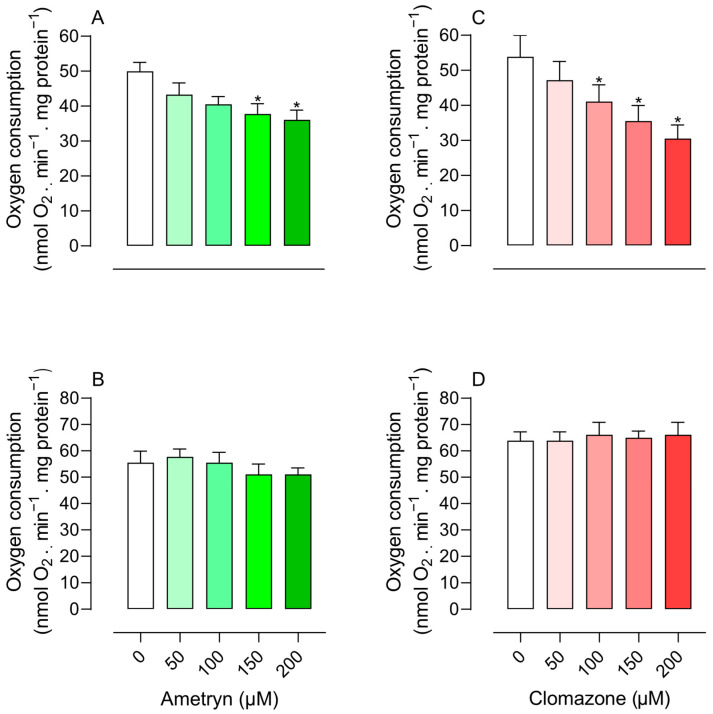
Effect of ametryn (green gradient) and clomazone (red gradient) on state 3 respiration of mitochondria isolated from rat liver. (**A**,**B**) effect of ametryn in mitochondria energized with 5 mM glutamate plus 5 mM malate or 5 mM succinate (+50 nM rotenone), respectively; (**C**,**D**) effect of clomazone in mitochondria energized with 5 mM glutamate plus 5 mM malate or 5 mM succinate (+50 nM rotenone), respectively. Control values expressed in nmol O_2_ . min^−1^ . mg protein^−1^: (**A**) 49.98 ± 2.55, (**B**) 55.53 ± 4.34, (**C**) 53.87 ± 6.11, and (**D**) 63.86 ± 3.38. Values represent the mean ± SEM of four experiments with different mitochondrial preparations. * Significantly different from the control, without the addition of herbicides (*p* < 0.05).

Mitochondrial oxygen consumption was measured in the presence of varying concentrations of AMT and CLZ (50–200 µM). The parameters evaluated included state 3 respiration (oxygen consumption in the presence of respiratory substrate and ADP) and state 4 respiration (oxygen consumption following ADP depletion). At concentrations of 150 and 200 µM, AMT reduced state 3 respiration in mitochondria energized with glutamate and malate (complex I electron donors) (Figure 1A). AMT did not affect state 3 respiration in mitochondria energized with succinate (electron donor of complex II) (Figure 1B).

**Figure 2:**
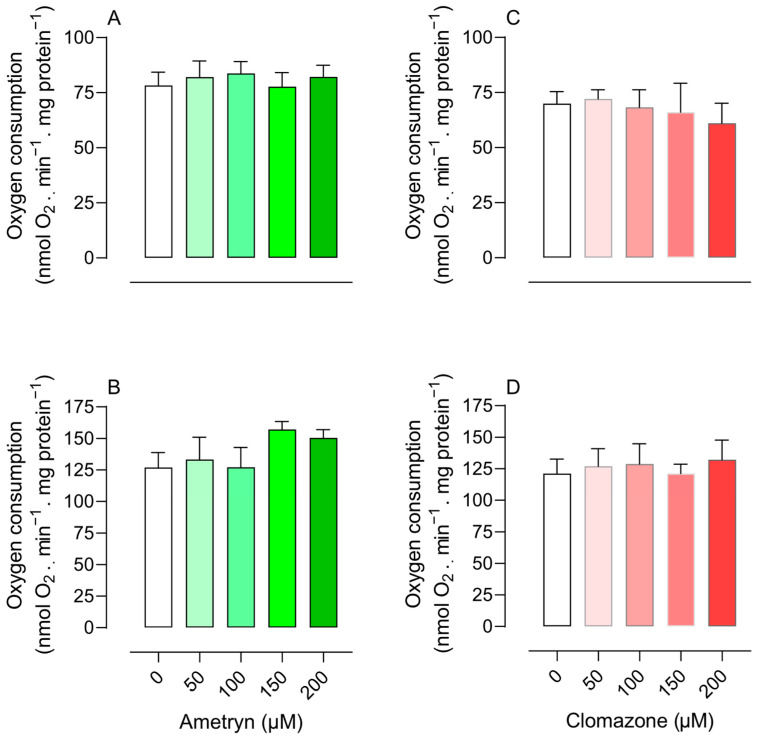
Effect of ametryn (green gradient) and clomazone (red gradient) on state 4 respiration of mitochondria isolated from rat liver. (**A**,**B**) effect of ametryn in mitochondria energized with 5 mM glutamate plus 5 mM malate or 5 mM succinate (+50 nM rotenone), respectively; (**C**,**D**) effect of clomazone in mitochondria energized with 5 mM glutamate plus 5 mM malate or 5 mM succinate (+50 nM rotenone), respectively. Control values expressed in nmol O_2_ . min^−1^ . mg protein^−1^: (**A**) 4.70 ± 0.36, (**B**) 7.63 ± 0.71, (**C**) 4.20 ± 0.32, and (**D**) 7.28 ± 0.68. Values represent the mean ± SEM of four experiments with different mitochondrial preparations.

The herbicide CLZ significantly inhibited state 3 respiration starting at a concentration of 100 µM in mitochondria energized with glutamate plus malate (Figure 1C) and did not affect oxygen consumption in mitochondria energized with succinate (Figure 1D). The compounds had no effect on state 3 respiration in mitochondria energized with TMPD-ascorbate (electron donor of complex IV). The compounds did not stimulate state 4 respiration, suggesting that they do not act as uncouplers (Figure 2).

The data shown in Table 1 reveal that the RCR was abolished only at higher concentration of AMT (200 μM) and at 150 and 200 μM of CLZ using glutamate plus malate (complex I substrates). AMT also abolished RCR at higher tested concentration (200 μM), while CLZ had no significant effect on the RCR of mitochondria energized with succinate (complex II substrate).

### 3.2. Effect of AMT and CLZ on Mitochondrial Membrane Potential (Δψ)

**Figure 3:**
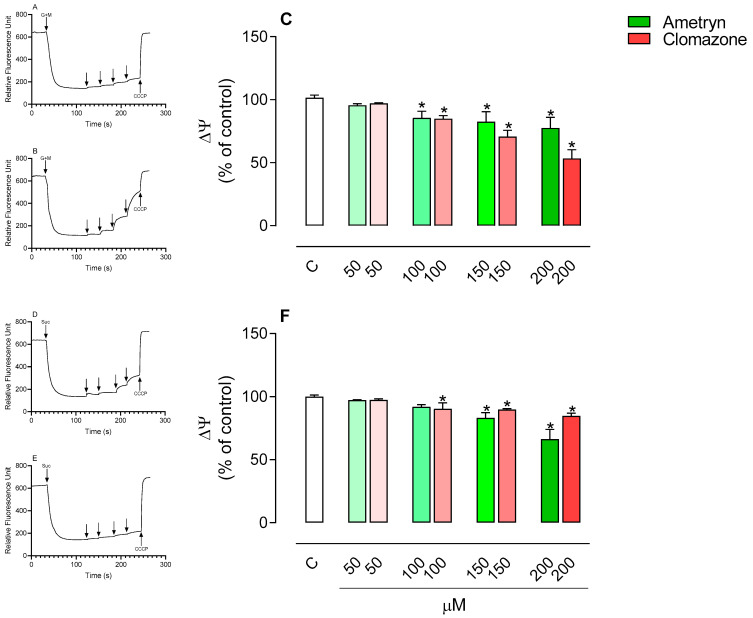
Effects of ametryn and clomazone on membrane potential in mitochondria isolated from rat liver energized with (**C**) 5 mM glutamate plus 5 mM malate and (**F**) 5 mM succinate (+50 nM rotenone). Traces (**A**,**B**) are representative of experiments performed with mitochondria energized with glutamate plus malate and traces (**D**,**E**) are representative of mitochondria energized with succinate. Values represent the mean ± SEM of four experiments with different mitochondrial preparations. * Significantly different from the control (*p* < 0.05). C = control, without the addition of herbicides, obtained with addition of 1 μM CCCP.

Figure 3A–C shows the effects of the tested herbicides on the membrane potential of mitochondria energized with glutamate plus malate. AMT and CLZ significantly reduced the mitochondrial membrane potential at concentrations of 100, 150, and 200 µM.

In mitochondria energized with succinate, AMT significantly reduced the mitochondrial membrane potential at concentrations of 150 and 200 µM, while CLZ showed an effect only at a concentration of 200 µM (Figure 3D–F).

### 3.3. Effect of AMT and CLZ on Mitochondrial ATP Levels

**Figure 4:**
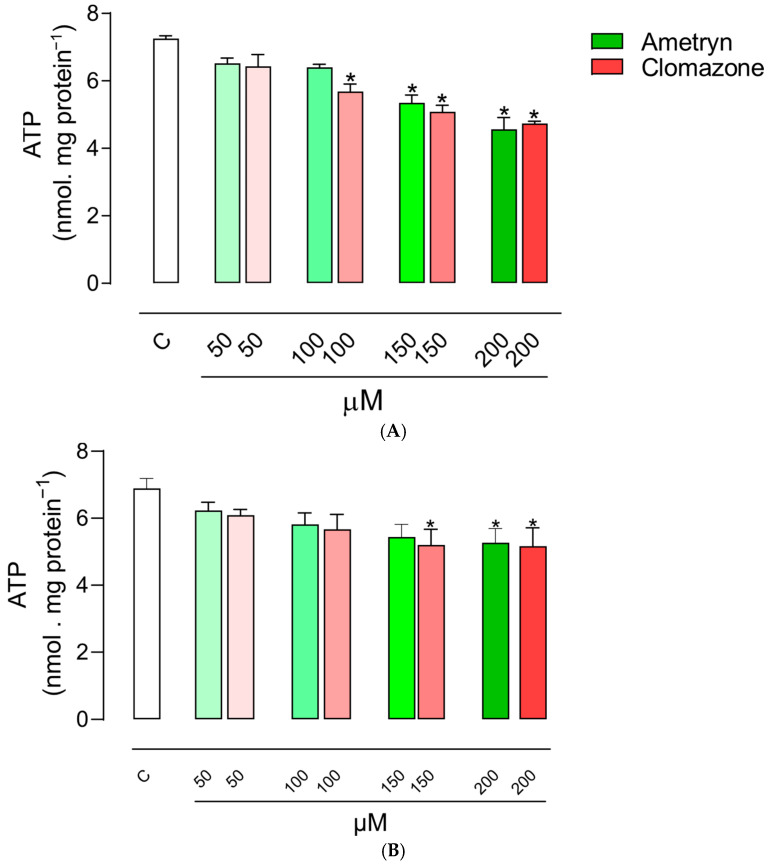
Effects of ametryn and clomazone on ATP concentration in mitochondria isolated from rat liver: (**A**) mitochondria energized with 5 mM glutamate plus 5 mM malate and (**B**) mitochondria energized with 5 mM succinate (+50 nM rotenone). Control values expressed in nmol. mg protein^−1^: 7.26 ± 0.08 or 6.89 ± 0.30 for malate + glutamate- or succinate-supported respiration, respectively. Values represent the mean ± SEM of four experiments with different mitochondrial preparations. * Significantly different from the control (*p* < 0.05). C = control, without the addition of herbicides.

The effects of the tested herbicides on ATP concentration in mitochondria energized with glutamate plus malate are shown in Figure 4A. AMT significantly reduced the mitochondrial ATP concentration at concentrations of 150 and 200 µM, while CLZ exhibited an inhibitory effect on ATP production starting at a concentration of 100 µM. The herbicide AMT caused significant changes in the ATP concentration in mitochondria energized with succinate only at 200 µM, while CLZ significantly reduced the ATP concentration from 150 µM (Figure 4B).

### 3.4. Influence of AMT and CLZ on Mitochondrial Enzymatic Activities

**Figure 5:**
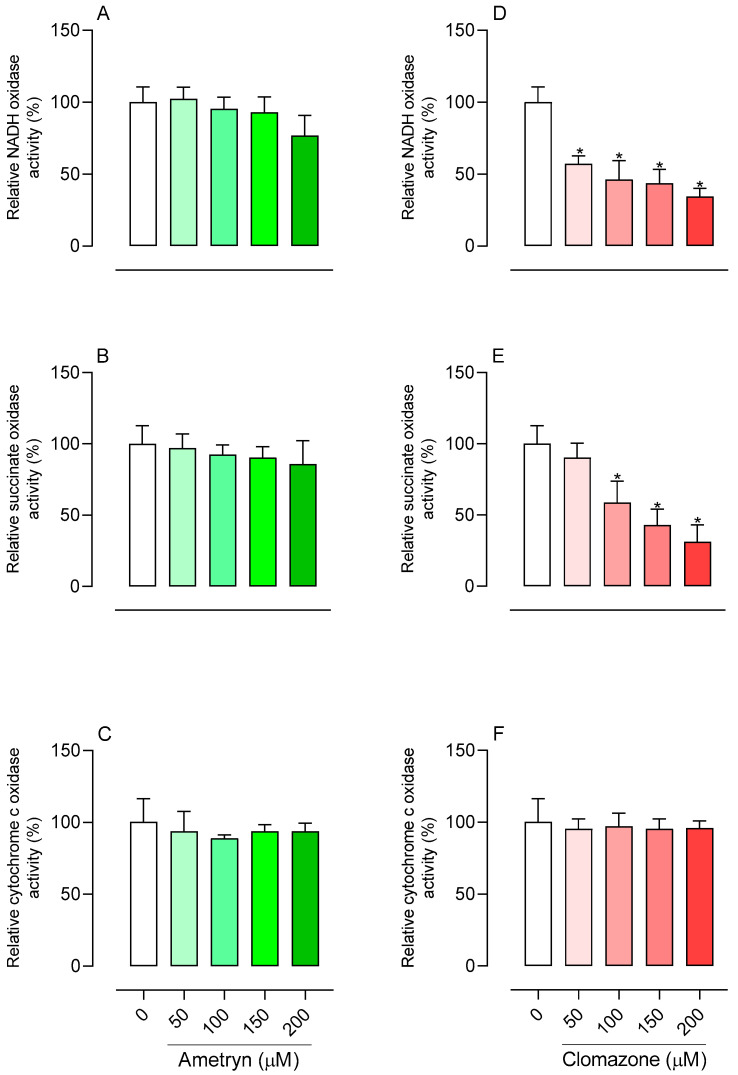
Effects of ametryn (green gradient) and clomazone (red gradient) on mitochondrial oxidase activities. Enzymatic activity is presented as a percentage relative to control. (**A**,**D**) NADH oxidase; (**B**,**E**) Succinate oxidase; (**C**,**F**) cytochrome *c* oxidase. Specific activities in the control were as follows: NADH oxidase, 24.76 ± 3.21 nmol O_2_ . min^−1^ . mg protein^−1^; succinate oxidase, 42.67 ± 8.55 nmol O_2_ . min^−1^ . mg protein^−1^; cytochrome c oxidase, 61.79 ± 5.22 nmol O_2_ . min^−1^ . mg protein^−1^. Values represent the mean ± SEM of six experiments with different mitochondrial preparations. * Significantly different from the control (*p* < 0.05). C = control, without the addition of herbicides.

NADH-mediated respiration (NADH oxidase activity) was not affected by AMT at any concentration tested (Figure 5A), and succinate-mediated respiration (succinate oxidase activity) was likewise unaffected (Figure 5B). In contrast, CLZ markedly inhibited NADH-driven respiration at all concentrations evaluated (Figure 5D) and reduced succinate-driven respiration at concentrations ≥ 100 µM (Figure 5E). The herbicides had no effect on TMPD and ascorbate-induced respiration, which drives cytochrome c–oxidase activity (Figure 5C,F).

**Figure 6:**
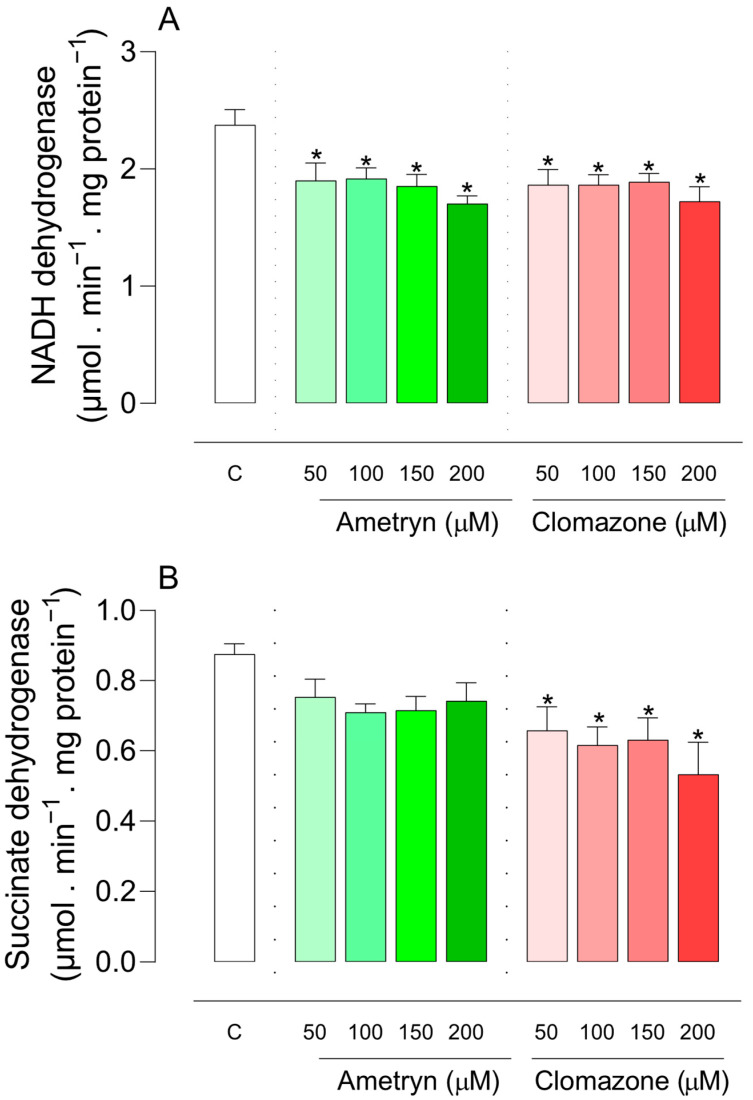
Effects of ametryn (green gradient) and clomazone (red gradient) on NADH (**A**) and succinate dehydrogenase (**B**) enzyme activities. Specific activities in the control were as follows: (**A**) 2.38 ± 0.13 μmol. min^−1^ . mg protein^−1^; (**B**) 0.88 ± 0.03 μmol. min^−1^ . mg protein^−1^. Values represent the mean ± SEM of five experiments with different mitochondrial preparations. * Significantly different from the control (*p* < 0.05). C = control, without the addition of herbicides.

Regarding the effect of herbicides on the activity of NADH dehydrogenase, an enzyme of complex I in the mitochondrial respiratory chain, an inhibitory effect was observed at all tested concentrations of AMT or CLZ (Figure 6A).

The herbicide AMT did not cause significant changes in the activity of the enzyme succinate dehydrogenase, while CLZ presented an inhibitory effect at all tested concentrations (Figure 6B).

### 3.5. Effects of AMT and CLZ on F_o_F_1_-ATPase Activity

**Figure 7:**
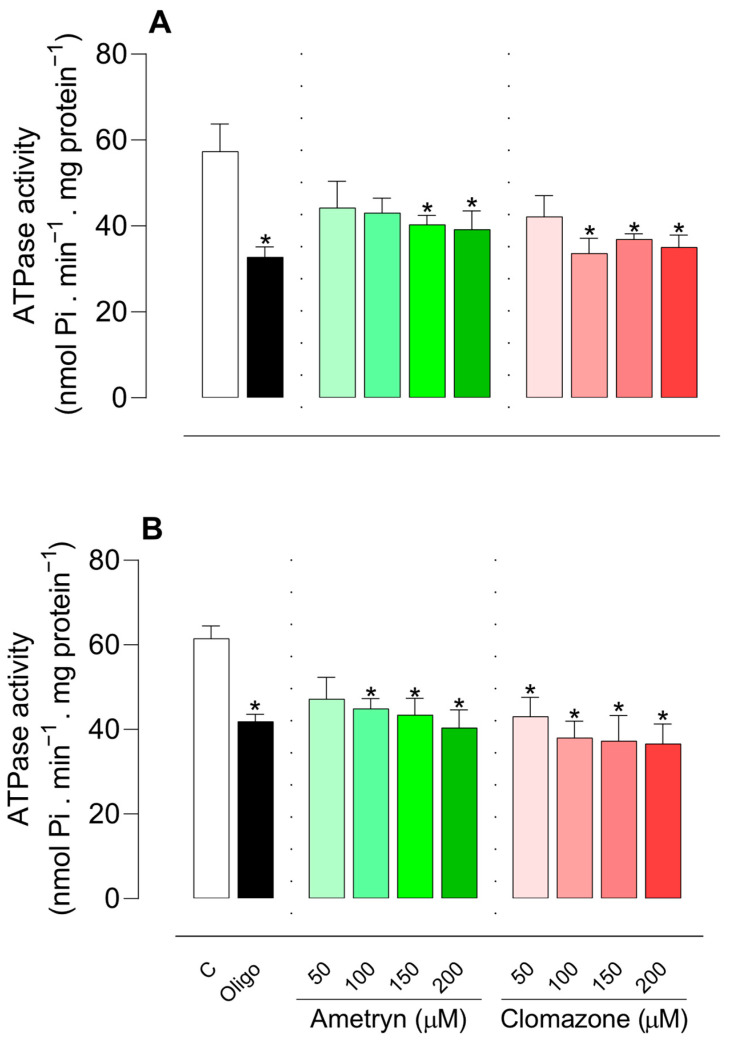
Effects of ametryn (green gradient) and clomazone (red gradient) on ATPase activity in intact-uncoupled mitochondria in the presence of CCCP (**A**) and in freeze–thawing-disrupted rat liver mitochondria (**B**). Specific activities in control were as follows: (**A**) 57.34 ± 6.42 nmol Pi. min^−1^ . mg protein^−1^; (**B**) 61.51 ± 2.99 nmol Pi. min^−1^ . mg protein^−1^. Values represent the mean ± SEM of five experiments with different mitochondrial preparations. * Significantly different from the control (*p* < 0.05). C = control, without the addition of herbicides. Oligo: oligomycin 1 μg/mL.

The effects of AMT and CLZ on F_o_F_1_-ATPase activity were assessed in intact-uncoupled mitochondria in the presence of CCCP and in freeze–thaw-disrupted mitochondria, as shown in Figure 7A,B, respectively. The ATPase activity of uncoupled mitochondria was decreased by AMT at concentrations of 150 and 200 µM, while CLZ decreased the enzyme activity from 100 µM (Figure 7A). In disrupted mitochondria, the effects were more pronounced, with AMT decreasing ATPase activity from 100 µM and CLZ in all concentrations tested (Figure 7B). 

**Figure 8:**
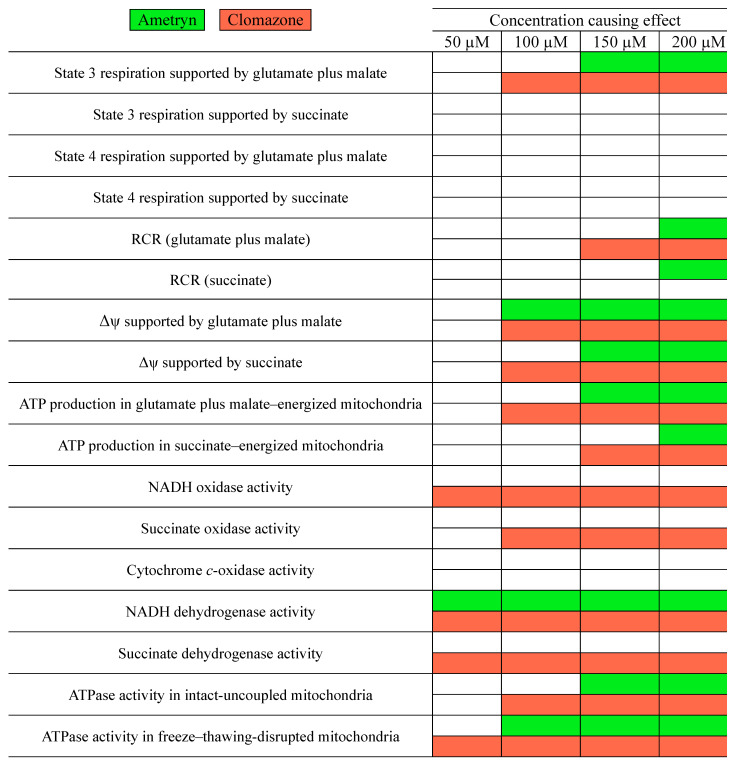
Summary of the effects of ametryn and clomazone on the bioenergetics of rat liver mitochondria. RCR = state 3 respiration rate/state 4 respiration rate. Δψ = mitochondrial membrane potential. ATP = adenosine triphosphate. NADH = nicotinamide adenine dinucleotide (reduced form). ATPase = ATP synthase operating in the reverse direction (ATP hydrolysis).

The overall effects of AMT and CLZ on parameters related to mitochondrial bioenergetics are presented in Figure 8.

## 4. Discussion

AMT and CLZ exposure have been associated with hepatotoxic effects in both aquatic and terrestrial vertebrates. Chronic oral exposure to sub-lethal doses of AMT in Wistar rats resulted in genotoxic effects, oxidative stress, hepatic fibrosis, and downregulation of gap junction proteins, indicating hepatocellular injury and epithelial-to-mesenchymal transition [46]. Moreover, a rare but fatal case of human poisoning by ingestion of an AMT-containing herbicide formulation (Gesapax^®^) was reported in Japan, where forensic toxicological analysis confirmed high concentrations of AMT in multiple tissues, corroborating its systemic absorption and acute lethality [70]. CLZ has demonstrated significant developmental and biochemical toxicity in zebrafish (*Danio rerio*) embryos and larvae. Exposure to CLZ concentrations as low as 36 µg/L resulted in a significant reduction in swim bladder inflation, an essential developmental process for buoyancy and locomotion, which, when impaired, can compromise larval survival [71]. Additionally, CLZ exposure was associated with a dose-dependent inhibition of acetylcholinesterase activity, indicating potential neurotoxicity, and with increased levels of hepatic biomarkers such as aspartate aminotransferase, alanine aminotransferase and alkaline phosphatase, revealing hepatotoxic effects [71].

Several literature reports have linked mitochondrial dysfunction to the toxic effects of pesticides observed in the liver [72,73,74,75,76,77]. Thus, to evaluate mitochondrial involvement in the hepatotoxicity associated with AMT, a *s*-triazine herbicide, and CLZ, a herbicide belonging to the class of isoxazolidinones, the present study assessed their effects on the bioenergetics of mitochondria isolated from rat liver. The effects of AMT and CLZ on mitochondrial respiration were monitored through oxygen consumption. AMT inhibited state 3 mitochondrial respiration in mitochondria energized with glutamate plus malate, with significant inhibition observed at concentrations starting from 150 µM. CLZ also affected mitochondria energized by glutamate plus malate at concentrations starting from 100 µM, with no impact on mitochondria energized by succinate. To evaluate the effect of AMT and CLZ as uncoupling agents, the state 4 respiration in mitochondria energized by glutamate plus malate or succinate was evaluated. The herbicides did not stimulate state 4 respiration under the conditions tested, which suggests a limited uncoupling effect; however, these findings do not exclude the possibility that mild or condition-dependent uncoupling could occur through alternative mechanisms.

In glutamate-plus-malate-supported respiration, the RCR, a parameter of mitochondrial function indicating the degree of coupling between the oxidation of respiratory substrates and ATP production [65], was significantly decreased in the presence of 200 µM AMT or 150 and 200 µM CLZ. These effects are shown in Table 1. On the other hand, the RCR for succinate-supported respiration was significantly decreased only in the presence of 200 µM AMT in the assay mixture, although the herbicide did not significantly inhibit respiration with this substrate.

AMT and CLZ decreased the membrane potential in mitochondria energized with glutamate plus malate from a concentration of 100 μM and succinate from 150 μM. Additionally, the ATP synthesis was significantly inhibited from 150 μM of AMT and 100 μM of CLZ in mitochondria energized with glutamate plus malate and at 200 μM of AMT and concentrations from 150 μM of CLZ in mitochondria energized with succinate. The lack of significant changes in state 3 and 4 respiration during complex II-linked respiration despite the observed decrease in membrane potential and ATP synthesis may reflect a mild uncoupling effect, where proton conductance is slightly increased. Such partial uncoupling can reduce the proton motive force and impair ATP synthesis without producing detectable changes in oxygen consumption rates [78,79]. These results, together with the inhibition detected in state 3 respiration, indicate that the herbicides could interfere with the electron transport of the respiratory chain or inhibit the oxidative phosphorylation, which led us to conduct additional assays to investigate these effects.

To elucidate the inhibitory effects of AMT and CLZ on NADH, succinate and cytochrome c oxidases assays were carried out using disrupted mitochondria. This approach allows assessment of electron transport through the respiratory chain independently of the intact membrane, phosphorylation activity, or other processes dependent on membrane potential, enabling identification of the specific site of respiratory chain inhibition. NADH oxidase activity was not significantly affected by AMT, whereas CLZ consistently inhibited this activity at all tested concentrations. Similarly, succinate oxidase activity was unaffected by AMT but was significantly inhibited by CLZ. No effect on cytochrome c oxidase was observed. These results indicate that, under the tested conditions, AMT does not appear to compromise electron flow through complexes I, II, or IV, whereas the inhibitory effect of CLZ on mitochondrial respiration involves both complexes I and II.

Because NADH- and succinate-driven oxidase activities depend not only on complexes I or II but also on downstream electron transport through complexes III and IV, we next examined the direct effects of the compounds on the enzymatic activities of complexes I and II. NADH:ubiquinone oxidoreductase or NADH dehydrogenase is the first proton-pumping enzyme of the mitochondrial respiratory chain and is considered the most susceptible to chemically induced dysfunction. Succinate dehydrogenase, in turn, is both a tricarboxylic acid (Krebs cycle) enzyme and the catalytic core of complex II of the respiratory chain [80]. AMT and CLZ inhibited NADH dehydrogenase activity in complex I, whereas only CLZ inhibited succinate dehydrogenase activity in complex II. These findings suggest that although both herbicides interfere with complex I, AMT exerts a more restricted action limited to NADH dehydrogenase, without affecting succinate dehydrogenase or downstream oxidase activities. In contrast, the broader inhibitory profile of CLZ, which targets both NADH dehydrogenase and succinate dehydrogenase, is consistent with its more pronounced effect on mitochondrial respiration. This differential behavior may indicate distinct binding sites or mechanisms of interaction with the complexes, reflecting a narrower specificity for AMT and a wider range of interference for CLZ. Although our study focused on complexes I and II, which are critical for NADH- and succinate-linked respiration, complex III activity was not directly assessed. Therefore, we cannot exclude the possibility that AMT and CLZ may also affect complex III, potentially contributing to the observed reductions in membrane potential and ATP production.

ATP synthase is a remarkable molecular motor crucial for generating ATP and sustaining mitochondrial function, and its malfunction has been linked to various pathological conditions [81]. ANT is a crucial part of the mitochondrial machinery of ATP production. Due to its involvement in both physiological (adenine nucleotide exchange) and pathological (mitochondrial permeability transition formation/regulation and cell death) mitochondrial processes, ANT is a key target for drug-induced toxicity [82]. To verify if AMT and CLZ could also inhibit ATP synthase and/or ANT, we evaluated its effects in the activity of ATPase using intact-uncoupled and freeze–thawing-disrupted mitochondria with an excess of ATP, a condition that drives the enzyme to operate in the reverse direction, hydrolyzing ATP. In intact-uncoupled mitochondria, the velocity of ATP hydrolysis depends on both ATPase activity and the rate of ADP/ATP exchange by ANT. In freeze–thawing-disrupted mitochondria, the velocity of ATP hydrolysis depends exclusively on ATPase activity, since ATP has free access to the enzyme [68].

Our results showed that herbicides present a more significant inhibition of ATPase activity in freeze–thawing-disrupted mitochondria than intact-uncoupled mitochondria, which indicates that both compounds more specifically inhibit F_o_F_1_-ATPase than ANT. This result is in line with that of Binukumar et al. (2010) [73], who reported that chronic exposure to the organophosphate pesticide dichlorvos resulted in significant impairment of hepatic energy metabolism, particularly inhibiting mitochondrial ATP synthase activity and Bizerra et al. (2018) [75] that observed that imidacloprid, a neonicotinoid pesticide, can directly affect the bioenergetics of liver mitochondria, interfering with F_o_F_1_ ATP synthase activity and impairing ATP production.

The involvement of mitochondrial dysfunction in triazines toxicity was described in different tissues. For instance, Thompson et al. (1974) [55] described the effect inhibitor of AMT and other triazines on the state 3 respiration of isolated rat liver mitochondria. Hase et al. [83] reported that atrazine inhibited state 3 respiration in sperm mitochondria. Additionally, incubation of liver mitochondria or skeletal muscle L6 cells with atrazine resulted in inhibition of mitochondrial complexes I, II, and III, as well as oxygen consumption [84]. Karadayian et al. (2022) [85] further demonstrated that atrazine could inhibit complex I–III activity in striatal mitochondria. Similarly, the literature data show that CLZ can damage animal cell mitochondria, especially the electron transport chain [30]. A study by Fidelis et al. (2024) [86] using mitochondria isolated from the thorax of honey bees (*Apis mellifera*) demonstrated that CLZ affected mitochondrial bioenergetics, leading to reduced ATP production. Similarly, Cestonaro et al. (2024) [87] showed that CLZ causes cytotoxic effect on THP-1 cells due to mitochondrial membrane depolarization. Therefore, the results of the present study corroborate previous literature reports and indicate that changes in mitochondrial bioenergetics may be related to AMT and CLZ hepatotoxicity.

There are two kinds of electron transport chain inhibitors, according to Boelsterli (2007) [80]: (1) those that bind to one of the chain’s components and prevent electron transport, and (2) those that actually promote electron flow through the first segments of the transport chain but, at some point, deflect the electron flow from its intended course by accepting the electron themselves. ATP synthase, essential for ATP production in oxidative phosphorylation, can be inhibited through different mechanisms depending on the class of inhibitors. For example: some inhibitors block proton passage through the F_o_ domain, preventing the proton flow required for enzyme rotation and, consequently, ATP synthesis; certain compounds act directly on the F_1_ domain, inhibiting the enzyme’s catalytic activity in converting ADP + Pi into ATP and some inhibitors can bind to allosteric sites, altering the ATP synthase conformation and reducing its activity [78]. Considering the difference in the structures of the compounds, while AMT has an ethylamino and an isopropylamino group, CLZ has a chlorobenzyl group bound to a N-O heterocycle called isoxazole, which can influence the polarity and reactivity of the molecules, future investigations including structural docking studies or inhibitor competition assays could elucidate the molecular interactions of AMT and CLZ with mitochondrial respiratory chain complexes and ATP synthase.

Similarly to our findings, Yamano and Morita (1995) [88] reported that several pesticides directly impaired mitochondrial function in isolated rat liver mitochondria, supporting the notion that xenobiotics can target oxidative phosphorylation and contribute to hepatocellular injury. Considering the complexity of mitochondrial processes involved in oxidative phosphorylation and their sensitivity to xenobiotic interference, approaches based on ordinary differential equations (ODEs) such as the one proposed by Nave (2020) [89], which modifies semi-analytical techniques for solving ODE systems, may offer useful tools to simulate and predict alterations in mitochondrial function in response to exposure to AMT and CLZ.

**Figure 9:**
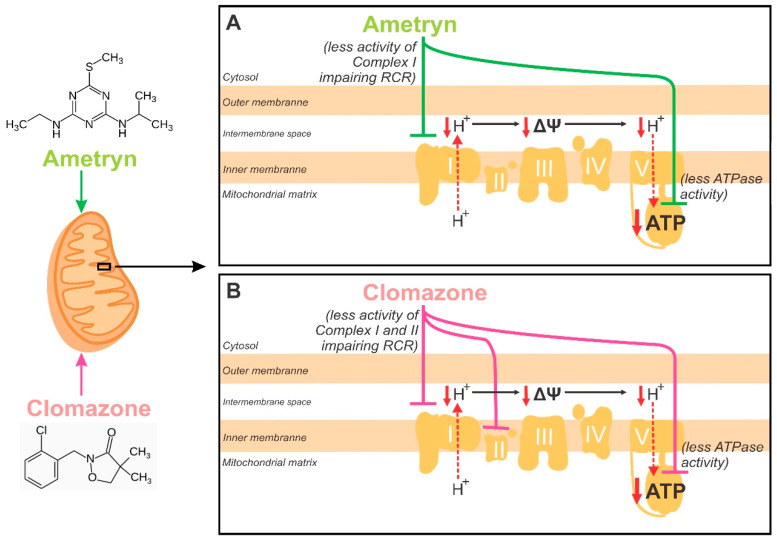
Proposed mechanisms underlying the bioenergetic impairment induced by ametryn (**A**) and clomazone (**B**) in mitochondria isolated from rat liver. Δψ = mitochondrial membrane potential. ATP = adenosine triphosphate. ATPase = ATP synthase.

Figure 9 shows the proposed mechanisms underlying the bioenergetic impairment induced by the herbicides. The primary effect of AMT is the inhibition of Complex I activity, which represents the upstream event leading to impaired electron transport, reduced proton pumping across the inner mitochondrial membrane, and consequently a decrease in Δψ. These upstream alterations secondarily impair the RCR and reduce the driving force for ATP synthase activity, culminating in lower ATP production as a downstream effect. In contrast, CLZ exerts its primary effects by simultaneously impairing both Complex I and Complex II activities, which diminishes electron flux through the electron transport chain at two entry points. This upstream inhibition reduces proton translocation and compromises Δψ, thereby impairing RCR. As a downstream consequence, ATP synthase activity is also reduced, leading to decreased mitochondrial ATP generation. Thus, while both herbicides converge on lowering Δψ and ATP production as final outcomes, AMT initiates toxicity primarily via Complex I, whereas CLZ targets both Complex I and II, suggesting a broader upstream impairment of mitochondrial bioenergetics.

Although ROS production was not directly measured in this study, the reductions observed in complexes I and II provide indirect evidence that ROS generation may be involved, warranting further investigation. While the concentrations of AMT and CLZ used here exceed typical environmental levels, these herbicides are frequently detected in water and food, leading to daily exposure in consumers. Moreover, populations living in or near treated agricultural areas, as well as pesticide applicators, are regularly exposed to these chemicals [2,90,91]. Therefore, our results indicate that both acute and chronic adverse effects on human health, particularly via hepatic mitochondrial dysfunction, should be taken into serious consideration. To further explore these effects under conditions that more closely mimic human exposure, ongoing experiments in our laboratory are assessing the impact of these herbicides on HepG2 liver cells.

A limitation of the present study is that we did not employ classical inhibitors of the mitochondrial electron transport chain, such as rotenone (complex I), antimycin A (complex III), or potassium cyanide (complex IV), which are traditionally used as positive controls to validate the site of action of xenobiotics on mitochondrial function [51,92]. Future investigations should incorporate such inhibitors, in combination with complementary approaches (e.g., high-resolution respirometry, purified complex assays, or genetic manipulation of mitochondrial proteins), to strengthen mechanistic conclusions and refine the identification of herbicides targets in mitochondrial bioenergetics.

## Figures and Tables

**Table 1:** Effect of ametryn and clomazone (50–200 µM) on the respiratory control ratio (RCR) using malate plus glutamate or succinate as oxidizable substrates.

	Malate Plus Glutamate	Succinate
	RCR	RCR
** Ametryn (µM) **		
0	6.25 ± 0.45	4.68 ± 0.15
50	5.76 ± 0.48	3.83 ± 0.27
100	5.21 ± 0.39	3.85 ± 0.33
150	5.03 ± 0.26	3.68 ± 0.53
200	4.48 ± 0.30 *	3.09 ± 0.19 *
** Clomazone (µM) ** 0	7.31 ± 0.31	4.91 ± 0.31
50	6.55 ± 0.24	4.70 ± 0.24
100	6.09 ± 0.45	4.73 ± 0.25
150	5.59 ± 0.54 *	4.94 ± 0.32
200	5.05 ± 0.18 *	4.11 ± 0.27

The results represent the mean ± SEM of four experiments with different preparations. * Significantly different from the control (*p* < 0.05). Control refers to the absence of ametryn and clomazone.

## Data Availability

Data will be made available on request.

## References

[1] Duke S.O. (1990). Overview of herbicide mechanisms of action. Environ. Health Perspect..

[2] Msibi S.S., Su L.J., Chen C.-Y., Chang C.-P., Chen C.-J., Wu K.-Y., Chiang S.-Y. (2023). Impacts of agricultural pesticide contamination: An integrated risk assessment of rural communities of Eswatini. Toxics.

[3] Montagner C.C., Sodré F.F., Acayaba R.D., Vidal C., Campestrini I., Locatelli M.A., Pescara I.C., Albuquerque A.F., Umbuzeiro G.A., Jardim W.F. (2019). Ten Years-Snapshot of the Occurrence of Emerging Contaminants in Drinking, Surface and Ground Waters and Wastewaters from São Paulo State, Brazil. J. Braz. Chem. Soc..

[4] Acayaba R.D., de Albuquerque A.F., Ribessi R.L., Umbuzeiro G.A., Montagner C.C. (2021). Occurrence of pesticides in waters from the largest sugar cane plantation region in the world. Environ. Sci. Pollut. Res..

[5] Silva M.C.S.C., Silva H.C.M.P., Teixeira K.P.S.B., Bedor D.C.G., Leal L.B., Santana D.P. (2020). Investigation into the occurrence of herbicide residues in the rivers of the Acaú-Goiana extractive reserve. Rev. Ibero-Am. Ciênc. Ambient..

[6] Echeverría-Sáenz S., Spínola-Parallada M., Soto A.C. (2021). Pesticides burden in neotropical rivers: Costa Rica as a case study. Molecules.

[7] US EPA—United States Environmental Protection Agency (2017). Ametryn: Preliminary Human Health Risk Assessment for Registration Review.

[8] Li Q.X., Hwang E.-C., Guo F. (2001). Occurrence of herbicides and their degradates in Hawaii’s groundwater. Bull. Environ. Contam. Toxicol..

[9] Li X.-S., Lu Z.-X., Lin M.-Z. (2006). Ametryn residues and its degradation dynamics in pineapple and soil. Chin. J. Eco-Agric..

[10] Programa de Análise de Resíduos de Agrotóxicos em Alimentos (PARA) (2024). Relatório dos Resultados das Análises de Amostras Monitoradas no Ciclo 2023; Brasília. https://www.gov.br/anvisa/pt-br/assuntos/agrotoxicos/programa-de-analise-de-residuos-em-alimentos/arquivos/relatorio-2013-para-2023.

[11] Wang Y.-J., Huang H.-L., Jiang L.-J., Zhuo H.-H., Liu H.-Z. (2010). Determination of ametryn residue in four plant-derived foods by gas chromatography coupled with solid phase extraction. Food Sci..

[12] Code of Federal Regulations (CFR) (2024). Title 40, §180.258—Ametryn; Tolerances for Residues.

[13] National Health and Medical Research Council (NHMRC) (2022). Australian Drinking Water Guidelines.

[14] Gunasekara A.S., Dela Cruz I.D., Curtis M.J., Claassen V.P.T., Jeerdema R.S. (2009). The behavior of clomazone in the soil environment. Pest Manag. Sci..

[15] Wang H., Ren W., Xu Y., Wang X., Ma J., Sun Y., Hu W., Chen S., Dai S., Song J. (2024). Long-term herbicide residues affect soil multifunctionality and the soil microbial community. Ecotoxicol. Environ. Saf..

[16] Marchesan E., Zanella R., Avila L.A., Camargo E.R., Machado S.L.O., Macedo V.R.M. (2007). Rice herbicide monitoring in two Brazilian rivers during the rice growing season. Sci. Agric..

[17] Brum A., Dotta G., Roumbedakis K., Gonçalves E.L.T., Garcia L.P., Garcia P., Scussel V.M., Martins M.L. (2013). Hematological and histopathological changes in silver catfish *Rhamdia quelen* (Siluriformes) exposed to clomazone herbicide in the Madre River, Santa Catarina State, Southern Brazil. J. Environ. Sci. Health B.

[18] Fournie M.L., Castillo L.E., Ramírez F., Moraga G., Ruepert C. (2018). Evaluación preliminar del área agrícola y su influencia sobre la calidad del agua en el Golfo Dulce, Costa Rica. Rev. Cienc. Ambient..

[19] Guarda P.M., Pontes A.M.S., Domiciano R.S., Gualberto L.S., Mendes D.B., Guarda E.A., Silva J.E.C. (2020). Assessment of ecological risk and environmental behavior of pesticides in environmental compartments of the Formoso River in Tocantins, Brazil. Arch. Environ. Contam. Toxicol..

[20] Seben D., Toebe M., Wastowski A.D., Hofstatter K., Volpatto F., Zanella R., Prestes O.D., Golombieski J.I. (2021). Water quality variables and emerging environmental contaminant in water for human consumption in Rio Grande do Sul, Brazil. Environ. Chall..

[21] Spurgeon D., Besien T., Armenise E., Hutt L., Kieboom N., Wilkinson H. (2021). Worst-Case Ranking of Organic Substances Detected in Groundwater and Surface Waters in England.

[22] Liu H., Li R., Hu W., Jian L., Huang B., Fan Y., Zhao Y., Zhang H., Khan K.S. (2024). Multi-medium residues and ecological risk of herbicides in a typical agricultural watershed of the Mollisols region, Northeast China. Sci. Total Environ..

[23] Health Canada (2024). Proposed Maximum Residue Limit PMRL2024-xx: Clomazone.

[24] Álvarez F., Arena M., Auteri D., Batista Leite S., Binaglia M., Castoldi A.F., Chiusolo A., Colagiorgi A., Colas M., EFSA (European Food Safety Authority) (2025). Peer review of the pesticide risk assessment of the active substance clomazone. EFSA J..

[25] University of Hertfordshire (2023). Clomazone (Ref: FMC 57020); Pesticide Properties DataBase (PPDB), Agriculture & Environment Research Unit (AERU), University of Hertfordshire. https://sitem.herts.ac.uk/aeru/ppdb/en/Reports/168.htm.

[26] Ohno Y., Miyajima A., Sunouchi M. (1998). Alternative methods for mechanistic studies in toxicology: Screening of hepatotoxicity of pesticides using freshly isolated and primary cultured hepatocytes and non-liver-derived cells, SIRC cells. Toxicol. Lett..

[27] EPA—United States Environmental Protection Agency (2018). Registration Review: Preliminary Human Health Risk Assessment for Ametryn. https://www.regulations.gov/document/EPA-HQ-OPP-2013-0249-0022.

[28] Crestani M., Menezes C., Glusczak L., Miron D.S., Spanevell R., Silveira A., Gonçalves F.F., Zanella R., Loro V.L. (2007). Effect of clomazone herbicide on biochemical and histological aspects of silver catfish (*Rhamdia quelen*) and recovery pattern. Chemosphere.

[29] Cattaneo R., Moraes B.S., Loro V.L., Pretto A., Menezes C., Sartori G.M.S., Clasen B., Avila L.A., Marchesan E., Zanella R. (2012). Tissue biochemical alterations of *Cyprinus carpio* exposed to commercial herbicide containing clomazone under rice-field conditions. Arch. Environ. Contam. Toxicol..

[30] Fagundes M.Z., Gonçalves M.A., Soares M.P., Martins M.L., Zanella R., Riet-Correa F., Anjos B.L. (2015). Clinicopathological and toxicological aspects of poisoning by the clomazone herbicide in sheep. Small Rumin. Res..

[31] Oliveira C.R., Fraceto L.F., Rizzi G.M., Salla R.F., Abdalla F.C., Costa M.J., Silva-Zacarin E.C. (2016). Hepatic effects of the clomazone herbicide in both its free form and associated with chitosan-alginate nanoparticles in bullfrog tadpoles. Chemosphere.

[32] Sevior D.K., Pelkonen O., Ahokas J.T. (2012). Hepatocytes: The powerhouse of biotransformation. Int. J. Biochem. Cell Biol..

[33] Beier J.I., Luo J., Vanderpuye C.M., Brizendine P., Muddasani P., Bolatimi O., Heinig S.A., Ekuban F.A., Siddiqui H., Ekuban A. (2025). Environmental Pollutants, Occupational Exposures, and Liver Disease. Semin. Liver Dis..

[34] Tolman K.G., Dalpiaz A.S., Kaplowitz N., DeLeve L.D. (2013). Occupational and Environmental Hepatotoxicity. Drug-Induced Liver Disease.

[35] VoPham T., Bertrand K.A., Hart J.E., Laden F., Brooks M.M., Yuan J.M., Talbott E.O., Ruddell D., Chang C.H., Weissfeld J.L. (2017). Pesticide exposure and liver cancer: A review. Cancer Causes Control.

[36] Fenton S.E., Ducatman A., Boobis A., DeWitt J.C., Lau C., Ng C., Smith J.S., Roberts S.M. (2021). Per- and polyfluoroalkyl substance toxicity and human health review: Current state of knowledge and strategies for informing future research. Environ. Toxicol. Chem..

[37] Jellali R., Jacques S., Essaouiba A., Gilard F., Letourneur F., Gakière B., Legallais C., Leclerc E. (2021). Investigation of steatosis profiles induced by pesticides using liver organ-on-chip model and omics analysis. Food Chem. Toxicol..

[38] Ramachandran A., Visschers R.G.J., Duan L., Akakpo J.Y., Jaeschke H. (2018). Mitochondrial dysfunction as a mechanism of drug-induced hepatotoxicity: Current understanding and future perspectives. J. Clin. Transl. Res..

[39] Mihajlovic M., Vinken M. (2022). Mitochondria as the target of hepatotoxicity and drug-induced liver injury: Molecular mechanisms and detection methods. Int. J. Mol. Sci..

[40] Dykens J.A., Jamieson J.D., Marroquin L.D., Nadanaciva S., Xu J.J., Dunn M.C., Smith A.R., Will Y. (2008). In vitro assessment of mitochondrial dysfunction and cytotoxicity of nefazodone, trazodone and buspirone. Toxicol. Sci..

[41] Leung M.C.K., Meyer J.N. (2019). Mitochondria as a target of organophosphate and carbamate pesticides: Revisiting common mechanisms of action with new approach methodologies. Reprod. Toxicol..

[42] Norat P., Soldozy S., Sokolowski J.D., Gorick C.M., Kumar J.S., Chae Y., Yağmurlu K., Prada F., Walker M., Levitt M.R. (2020). Mitochondrial dysfunction in neurological disorders: Exploring mitochondrial transplantation. Regen. Med..

[43] Chen P., Yao L., Yuan M., Wang Z., Zhang Q., Jiang Y., Li L. (2024). Mitochondrial dysfunction: A promising therapeutic target for liver diseases. Genes Dis..

[44] Nickel A., Kohlhaas M., Maack C. (2014). Mitochondrial reactive oxygen species production and elimination. J. Mol. Cell. Cardiol..

[45] Chen L., Zhang X., Zhou W., Qiao Q., Liang H., Li G., Wang J., Cai F. (2013). The interactive effects of cytoskeleton disruption and mitochondria dysfunction lead to reproductive toxicity induced by microcystin-LR. PLoS ONE.

[46] Santos T., Cancian G., Neodini D.N.R., Mano D.R.S., Capucho C., Predes F.S., Barbieri R., Oliveira C.A., Pigoso A.A., Dolder H. (2015). Toxicological evaluation of ametryn effects in Wistar rats. Exp. Toxicol. Pathol..

[47] Moura M.A.M., Oliveira R., Jonsson C.M., Domingues I., Soares A.M.V.M., Nogueira A.J.A. (2018). The sugarcane herbicide ametryn induces oxidative stress and developmental abnormalities in zebrafish embryos. Environ. Sci. Pollut. Res. Int..

[48] Santi A., Menezes C., Duarte M.M., Leitemperger J., Lópes T., Loro V.L. (2011). Oxidative stress biomarkers and acetylcholinesterase activity in human erythrocytes exposed to clomazone (in vitro). Interdiscip. Toxicol..

[49] Menezes C.C., Loro V.L., Fonseca M.B., Cattaneo R., Pretto A., Miron D.S., Santi A. (2011). Oxidative parameters of *Rhamdia quelen* in response to commercial herbicide containing clomazone and recovery pattern. Pestic. Biochem. Physiol..

[50] Teodoro J.S., Machado I.F., Palmeira C.M., Rolo A.P., Palmeira C.M., Rolo A.P. (2021). Determination of oxidative phosphorylation complexes activities. Mitochondrial Regulation.

[51] Hatefi Y. (1985). The mitochondrial electron transport and oxidative phosphorylation system. Annu. Rev. Biochem..

[52] Singer T.P. (1974). Determination of the activity of succinate, NADH, choline, and alpha-glycerophosphate dehydrogenases. Methods Biochem. Anal..

[53] Rich P.R., Maréchal A. (2010). The mitochondrial respiratory chain. Essays Biochem..

[54] Sun F., Huo X., Zhai Y., Wang A., Xu J., Su D., Bartlam M., Rao Z. (2005). Crystal structure of mitochondrial respiratory membrane protein complex II. Cell.

[55] Thompson O.C., Truelove B., Davis D.E. (1974). Effects of triazines on energy relations of mitochondria and chloroplasts. Weed Sci..

[56] Timossi P., Alves P. (2001). Effects of clomazone drift, sprayed alone or in mixture with ametryn, on the productive characteristics of Hamlin orange. Planta Daninha.

[57] Esqueda V.A., Altamirano L., Hernández Y., López A. (2006). Evaluation of the mixture ametryn + clomazone on sugarcane. Agron. Mesoam..

[58] Costa N.V., Gibbert A.M., Ferreira S.D., Canavessi H., Salvalaggio A.C. (2020). Strategies of chemical management for weed control in cassava. Rev. Ceres.

[59] Armas E.D., Monteiro R.T.R., Antunes P.M., Santos M.A.P.F., Camargo P.B., Abakerli R.B. (2007). Diagnóstico espaço-temporal da ocorrência de herbicidas nas águas superficiais e sedimentos do Rio Corumbataí e principais afluentes. Química Nova.

[60] Santos E.A., Correia N.M., Silva J.R.M., Velini E.D., Passos A.B.R.J., Durigan J.C. (2015). Herbicide detection in groundwater in Córrego Rico-SP watershed. Planta Daninha.

[61] CETESB—Companhia Ambiental do Estado do São Paulo (2021). Diagnóstico da Contaminação de Águas Superficiais, Subterrâneas e Sedimentos por Agrotóxicos.

[62] Li R., Hu W., Liu H., Huang B., Jia Z., Liu F., Zhao Y., Khan K.S. (2024). Occurrence, distribution and ecological risk assessment of herbicide residues in cropland soils from the Mollisols region of Northeast China. J. Hazard. Mater..

[63] Pedersen P.L., Greenawalt J.W., Reynafarje B., Hullihen J., Decker G.L., Soper J.W., Bustamante E. (1978). Preparation and characterization of mitochondria and submitochondrial particles of rat liver and liver-derived tissues. Methods in Cell Biology.

[64] Cain K., Skilleter D.N., Snell K., Mullock B. (1987). Preparation and use of mitochondria in toxicological research. Biochemical Toxicology.

[65] Chance B., Williams G.R. (1956). The respiratory chain and oxidative phosphorylation. Adv. Enzymol. Relat. Subj. Biochem..

[66] Zanotti A., Azzone G.F. (1980). Safranine as membrane potential probe in rat liver mitochondria. Arch. Biochem. Biophys..

[67] Lemasters J.J., Hackenbrock C.R. (1976). Continuous measurement and rapid kinetics of ATP synthesis in rat liver mitochondria, mitoplasts and inner membrane vesicles determined by firefly-luciferase luminescence. Eur. J. Biochem..

[68] Bracht A., Ishii-Iwamoto E.L., Salgueiro-Pagadigorria C.L., Bracht A., Ishii-Iwamoto E.L. (2003). Estudo do metabolismo energético em mitocôndrias isoladas de tecido animal. Métodos de Laboratório em Bioquímica.

[69] Fiske C.H., Subbarow Y. (1925). The colorimetric determination of phosphorus. J. Biol. Chem..

[70] Takayasu T., Sano Y., Nagai T., Saito K., Chiba T., Yoshida K., Ameno K. (2010). A fatal poisoning with the herbicide Gesapax: Ametryn and atrazine determination in human tissues. J. Anal. Toxicol..

[71] Miranda C.A., Peixoto P.V.L., Viriato C., Aggio L., Pereira L.C., Mingatto F.E. (2025). Toxicity of ametryn and clomazone in zebrafish: Environmentally relevant concentrations induce developmental and enzymatic alterations. Environ. Toxicol. Pharmacol..

[72] Yamano T., Morita S. (1993). Effects of pesticides on isolated rat hepatocytes, mitochondria, and microsomes. Arch. Environ. Contam. Toxicol..

[73] Binukumar B.K., Bal A., Kandimalla R., Sunkaria A., Gill K.D. (2010). Mitochondrial energy metabolism impairment and liver dysfunction following chronic exposure to dichlorvos. Toxicology.

[74] Liu Q., Wang Q., Xu C., Shao W., Zhang C., Liu H., Jiang Z., Gu A. (2017). Organochloride pesticides impaired mitochondrial function in hepatocytes and aggravated disorders of fatty acid metabolism. Sci. Rep..

[75] Bizerra P.F.V., Guimarães A.R.J.S., Maioli M.A., Mingatto F.E. (2018). Imidacloprid affects rat liver mitochondrial bioenergetics by inhibiting F_o_F_1_-ATP synthase activity. J. Toxicol. Environ. Health A.

[76] Miranda C.A., Guimarães A.R.J.S., Bizerra P.F.V., Mingatto F.E. (2020). Diazinon impairs bioenergetics and induces membrane permeability transition on mitochondria isolated from rat liver. J. Toxicol. Environ. Health A.

[77] Mirkhamidova P., Abduraxmonova M., Nishanbayev S., Mukhamedov G. (2024). Oxidative phosphorylation of rat liver mitochondria with intoxication of haloxyfop-r-methyl and indoxacarb pesticides. BIO Web Conf..

[78] Brand M.D., Nicholls D.G. (2011). Assessing mitochondrial dysfunction in cells. Biochem. J..

[79] Divakaruni A.S., Brand M.D. (2011). The regulation and physiology of mitochondrial proton leak. Physiology.

[80] Boelsterli U.A. (2007). Disruption of mitochondrial function and mitochondria mediated toxicity. Mechanistic Toxicology: The Molecular Basis of How Chemicals Disrupt Biological Targets.

[81] Althaher A.R., Alwahsh M. (2023). An overview of ATP synthase, inhibitors, and their toxicity. Heliyon.

[82] Oliveira P.J., Wallace K.B. (2006). Depletion of adenine nucleotide translocator protein in heart mitochondria from doxorubicin-treated rats. Relevance for mitochondrial dysfunction. Toxicology.

[83] Hase Y., Tatsuno M., Nishi T., Kataoka K., Kabe Y., Yamaguchi Y., Ozawa N., Natori M., Handa H., Watanabe H. (2008). Atrazine binds to F1F0-ATP synthase and inhibits mitochondrial function in sperm. Biochem. Biophys. Res. Commun..

[84] Lim S., Ahn S.Y., Song I.C., Chung M.H., Jang H.C., Park K.S., Lee K.U., Pak Y.K., Lee H.K. (2009). Chronic exposure to the herbicide, atrazine, causes mitochondrial dysfunction and insulin resistance. PLoS ONE.

[85] Karadayian A.G., Paez B., Bustamante J., Lores-Arnaiz S., Czerniczyniec A. (2023). Mitochondrial dysfunction due to in vitro exposure to atrazine and its metabolite in striatum. J. Biochem. Mol. Toxicol..

[86] Fidelis J.P.S., Miranda C.A., Nicodemo D., Mingatto F.E. (2024). Clomazone herbicide impairs bioenergetics in mitochondria isolated from the thorax of honey bees (*Apis mellifera* L.). Braz. J. Anim. Environ. Res..

[87] Cestonaro L.V., da Silva A.C.G., Garcia S.C., Valadares M.C., Arbo M.D. (2024). Mitochondrial impairment related to the immunotoxicity of the herbicides clomazone, glyphosate and sulfentrazone in THP-1 cells. Toxicol. Res..

[88] Yamano T., Morita S. (1995). Effects of pesticides on isolated rat hepatocytes, mitochondria, and microsomes II. Arch. Environ. Contam. Toxicol..

[89] Nave O.P. (2020). Modification of semi-analytical method applied system of ODE. Mod. Appl. Sci..

[90] Msibi S.S., Chen C.-Y., Chang C.-P., Chen C.-J., Chiang S.-Y., Wu K.-Y. (2021). High pesticide inhalation exposure from multiple spraying sources amongst applicators in Eswatini, Southern Africa. Pest Manag. Sci..

[91] Essandoh Y.E., Steiniche T., Xia C., Romanak K., Ogwang J., Mutegeki R., Wasserman M., Venier M. (2025). Tracking toxic chemical exposure in Uganda: Insights from silicone wristbands. Environ. Res..

[92] Nicholls D.G., Ferguson S.J. (2013). The respiratory chain. Bioenergetics.

